# MGMT gene promoter methylation by pyrosequencing method correlates volumetric response and neurological status in IDH wild-type glioblastomas

**DOI:** 10.1007/s11060-022-03999-5

**Published:** 2022-04-10

**Authors:** Tomohiro Hosoya, Masamichi Takahashi, Mai Honda-Kitahara, Yasuji Miyakita, Makoto Ohno, Shunsuke Yanagisawa, Takaki Omura, Daisuke Kawauchi, Yukie Tamura, Miyu Kikuchi, Tomoyuki Nakano, Akihiko Yoshida, Hiroshi Igaki, Yuko Matsushita, Koichi Ichimura, Yoshitaka Narita

**Affiliations:** 1 Department of Neurosurgery and Neuro-Oncology, National Cancer Center Hospital, 5-1-1, Tsukiji, Chuo-ku, Tokyo, 104-0045 Japan; 2 Department of Radiation Oncology, National Cancer Center Hospital, 5-1-1, Tsukiji, Chuo-ku, Tokyo, 104-0045 Japan; 3 Department of Pathology, National Cancer Center Hospital, 5-1-1, Tsukiji, Chuo-ku, Tokyo, 104-0045 Japan; 4 Department of Brain Disease Translational Research, Juntendo University Faculty of Medicine, Tokyo, Japan

**Keywords:** Glioblastoma, Volumetric analysis, MGMT methylation, Cutoff value

## Abstract

**Purpose:**

Although the usefulness of *O*^6^-methylguanine DNA methyltransferase (MGMT) promoter methylation analysis for predicting response to chemoradiotherapy and the prognosis of patients with glioblastoma has been widely reported, there is still no consensus regarding how to define MGMT promoter methylation percentage (MGMTpm%) cutoffs by pyrosequencing method. The aim of this study was to determine the optimal cutoff value of MGMT promoter methylation status using volumetric analysis focused on the tumor volume ratio (TVR) measured by MRI.

**Methods:**

This retrospective study included newly diagnosed IDH wild-type glioblastoma patients with residual tumor after surgery, followed by local radiotherapy with temozolomide. TVR was defined as the tumor volume at 6 months after the initial chemoradiotherapy administration divided by the tumor volume before the start of therapy. The mean MGMTpm% of 16 CpG islands (74–89) was analyzed using pyrosequencing. We statistically analyzed the correlation between MGMTpm%, TVR, and change in Karnofsky performance status.

**Results:**

The study included 44 patients with residual tumors. Thirteen (92.9%) of 14 patients with MGMTpm% ≥ 23.9% showed 50% or more volumetric response, leading to prolonged survival, and 17 (70.8%) of 24 patients with MGMTpm% < 8.2% had progressive disease after initial chemoradiotherapy administration. Three (50.0%) of six patients with MGMTpm% 8.2% to < 23.9% had stable disease or partial response.

**Conclusion:**

Evaluation of MGMTpm% by pyrosequencing is important in predicting the volumetric response and prognosis of glioblastoma patients with residual tumors.

## Introduction

Glioblastoma is the most aggressive malignant brain tumor in adults. The standard treatment for newly diagnosed glioblastoma is maximal safe resection and postoperative local radiotherapy with concomitant temozolomide followed by adjuvant temozolomide. However, the median survival and 2-year survival rates with the above standard treatment was reported to be 14.6 months and 26.5%, respectively [1].

Prognostic factors of glioblastoma are age, neurological status, preoperative Karnofsky performance status (KPS), extent of resection, and *O*^6^-methylguanine DNA methyltransferase (MGMT) promoter methylation. Many studies have reported MGMT promoter methylation as a prognostic factor for glioblastoma. The MGMT gene encodes a DNA repair enzyme that removes alkyl adducts from the *O*^6^ position of guanine in tumor DNA, which is damaged by alkylating agents such as temozolomide, exerting antitumor effects by impairing DNA replication. Therefore, MGMT expressed in tumor cells removes the guanine-alkyl group and attenuates the antitumor effect [2], while patients with MGMT promoter methylation have a better response to temozolomide and a better prognosis because of the suppression of MGMT in tumor cells. Furthermore, it has been reported that MGMT promoter methylation status is associated with prolonged survival in patients treated with radiotherapy alone [2, 3]. Thus, MGMT promoter methylation status is a prognostic and a predictive marker for temozolomide in glioblastomas, and evaluation of MGMT promoter methylation is considered important for predicting treatment efficacy and prognosis. The population of glioblastoma patients with MGMT promoter methylation was reported to be 25–47% in recent randomized clinical trials [1, 2, 4–8].

Pyrosequencing is a sequencing-based method that can analyze several CpG positions simultaneously and generates quantitative results for each analyzed CpG position individually with rapid parallel processing of a large number of samples [9]. There have been many reports for determining the cutoff value of MGMT promoter methylation status based on methylation percentage of CpG positions in the MGMT promoter gene. These reports were analyzed based on overall survival (OS) or progression-free survival (PFS). However, a cutoff value that discriminates MGMT promoter methylation status has not yet been determined.

Gross total resection was achieved in less than half of glioblastoma patients, and the response to temozolomide in patients with residual tumors is not fully understood. In this study, to obtain more specific and useful information about the response to temozolomide and the actual clinical course, we focused on the volume change of residual tumor and statistically examined the relationship between MGMT promoter methylation percentage (MGMTpm%) and response to temozolomide or survival.

## Materials and methods

### Patients

This retrospective study included newly diagnosed IDH wild-type glioblastoma patients who underwent surgery followed by local radiotherapy equivalent to 60 Gy and concomitant chemotherapy with temozolomide [1]. Approval for this study was obtained from the Internal Review Board of the National Cancer Center Hospital. Of the 350 patients with primary glioblastoma who underwent surgery and received temozolomide-based chemoradiotherapy at the National Cancer Center Hospital (5-1-1, Tsukiji, Chuo-ku, Tokyo, Japan) between September 2006 and December 2021, patients who had postoperative volumetrically measurable residual tumor tissue and received adjuvant temozolomide therapy for at least 6 months after the initial chemoradiotherapy administration were included in this study. Patients without measurable contrast lesions after gross total resection were excluded. We also excluded patients who received bevacizumab, nivolumab, procarbazine, and novo-TTF therapy in addition to temozolomide and those who underwent reoperation for tumor recurrence within 6 months. Clinical characteristics were also examined from each patient’s records.

### Tumor volume change ratio

The volume of contrast-enhancing lesions was calculated based on 1.5–3 T magnetic resonance imaging (MRI) scans captured at each time point: base line within 3 days after tumor resection and 6 months after initial chemoradiotherapy administration. The volume was calculated by multiplying the gross area of the contrast-enhanced lesions in each section by the slice thickness. The borders of the contrast-enhanced lesions were manually traced on MRI scans in a blinded fashion to the patient’s MGMTpm% results. Non-contrast areas within the contrast areas were measured as lesions, but obvious cystic lesions or postoperative extraction cavities were excluded from the measurement. Tumor volume ratio (TVR) was defined as the tumor volume at 6 months after the initial chemoradiotherapy administration divided by the tumor volume before the start of chemoradiotherapy.

### Molecular analysis

DNA was extracted from frozen tumor tissues or formalin-fixed paraffin-embedded (FFPE) tissues using a DNeasy Blood & Tissue Kit (Qiagen, Tokyo, Japan), and bisulfite modification of genomic DNA (500 ng) was performed using an EZ DNA methylation kit (Zymo Research, Orange, CA, USA). Pyrosequencing primers were designed to cover 16 CpG sites (CpG74–89) of the MGMT promoter gene [10]. Pyrosequencing of IDH1/2 and MGMT promoter genes was performed using PyroGold Q96 SQA Reagents and PyroMark Q96 software (version 2.5.7) on a pyrosequencing96 pyrosequencer (Qiagen, Tokyo, Japan) according to the manufacturer’s recommendations. The data were analyzed using PyroMark Q96 software, as described previously [10, 11]. The mean percentage of MGMT promoter methylation was calculated by averaging 16 CpG islands (74–89) and was analyzed by the pyrosequencing method as described previously [10].

### Statistical analysis

JMP ver.14 was used for all the statistical analyses, and statistical significance was set at p < 0.05. Volumetric assessment was performed 6 months after the start of chemoradiotherapy based on the Response Assessment in Neuro-Oncology (RANO) criteria [12]. Volumetric complete response (CR), partial response (PR), stable disease (SD), and progressive disease (PD) were defined as no residual tumor, ≥ 50% decrease, < 50% decrease to < 25% increase, and ≥ 25% increase in TVR, respectively. Multiple lesions were evaluated based on the total volume of each lesion. We classified two categories based on the TVR: (1) a CR/PR/SD group versus a PD group with a TVR cutoff value of 1.25 (25% volume increase or not), and (2) a CR/PR group versus SD/PD group with a TVR cutoff value of 0.5 (50% volume decrease or not). We used receiver operating characteristic (ROC) curve analysis to analyze the diagnostic value of MGMTpm% for TVR as a response to the therapy. The optimal cutoff point was defined as the point on the ROC curve nearest to the upper left corner of the graph, and the area under the curve (AUC) was used as an index of predictive accuracy. When adjacent points were detected on the graph, we selected the optimal cutoff point with an emphasis on specificity. Additionally, to analyze whether MGMT promoter methylation status with the cutoff value can be used to provide long-term prognostic stratification, survival was analyzed using the Kaplan–Meier curve and log-rank test. OS was defined as the time from the date of surgery to the date of death or the last follow-up date, and PFS was defined as the time from the date of surgery to the date of disease progression or the last follow-up date according to the RANO criteria. Patients who were still alive at the end of the observation period or who were lost to follow-up within the observation period were censored.

## Results

### Patient characteristics

Forty-four patients were eligible for this study, including 22 men (50.0%) and 22 women (50.0%), with a median age of 65 years (interquartile range [IQR] 55.3–73.8) (Table 1). There were 23 patients (52.3%) aged 65 years or more, and 21 patients (47.7%) were younger than 65 years. KPS ≥ 80 and KPS < 80 were 59.1% and 41.0%, respectively. The extent of resection was categorized into 90–99% and < 90% tumor removal and that of 90–99% removal or more was 19 patients (43.2%). The median Ki-67 staining index of each tumor was 28.3% (IQR 16.5–49.8%). The MGMT promoter was examined from 39 frozen samples and 5 FFPE tissues from 44 patients, and the median MGMTpm% was 5.5% (IQR 0.8–31.6%). The Mann–Whitney U test was used to examine the difference in TVR between the two groups, while the Kruskal–Wallis test was used to examine the difference in TVR between more than two groups. We also performed a multivariate logistic regression analysis to assess whether there were significant differences in TVR between age, KPS, and MGMTpm%, which were significant in the univariate analysis. In this analysis, TVR was categorized into binary dependent variables (1: better response with TVR ≤ 1.20; 0: poorer response with TVR > 1.20) by using the median TVR value of 1.20. Thereby, age ≥ 65 years (OR 8.51, 95% CI 1.27–57.31, p = 0.0277) and high-MGMTpm% (OR 38.37, 95% CI 3.05–482.61, p = 0.0048) showed significantly better treatment response.

**Table 1:** Baseline patient characteristics

Variables		No. of patients (%)	TVR at 6 months (median, IQR)	Univariate analysis	Multivariate analysis			
				p value^a^		OR	95% CI	p value^b^
All patients		44	1.19 (0.22–3.51)					
Age	Age ≥ 65	23 (52.3%)	0.33 (0.09–1.42)	0.0018		8.51	1.27–57.31	0.0277
	Age < 65	21 (47.7%)	2.79 (0.87–11.30)			1	Referent	
Sex	Men	22 (50.0%)	1.30 (0.28–3.93)	0.3914				
	Women	22 (50.0%)	1.01 (0.13–3.83)					
KPS	90	11 (25.0%)	3.04 (1.21–6.48)	0.0266	(KPS ≥ 80)	0.42	0.07–2.64	0.3554
	80	15 (34.1%)	1.42 (0.33–10.34)					
	70	12 (27.3%)	0.48 (0.30–2.35)		(KPS < 80)	1	Referent	
	60	4 (9.1%)	0.02 (0–0.08)					
	50	2 (4.6%)	1.03 (0.22–1.84)					
Lesion	Single	38 (86.4%)	1.14 (0.14–3.83)	0.3556				
	Multiple	6 (13.6%)	2.38 (0.48–5.37)					
Extent of removal	≥ 90%	19 (43.2%)	1.42 (0.13–10.34)	0.4844				
	< 90%	25 (56.8%)	0.82 (0.22–2.91)					
Pre-treatment RTV	≥ 2511 mm^3^	22 (50.0%)	1.14 (0.28–2.69)	0.7602				
	< 2511 mm^3^	22 (50.0%)	1.31 (0.12–10.82)					
Ki-67 staining index	≥ 28.3%	22 (50.0%)	0.36 (0.08–2.54)	0.0737				
	< 28.3%	22 (50.0%)	1.97 (0.50–6.43)					
MGMTpm%	≥ 23.9%	14 (31.8%)	0.12 (0–0.35)	0.0001		38.37	3.05–482.61	0.0048
	8.2–23.9%	6 (13.6%)	1.75 (0.47–3.83)			1.43	0.12–16.57	0.775
	< 8.2%	24 (54.6%)	2.52 (1.11–11.78)			1	Referent	

*RTV* residual tumor volume, *MGMTpm%* MGMT promoter methylation %, *TVR* tumor volume ratio, *IQR* interquartile range, *OR* odds ratio, *CI* confidence interval

^a^Mann Whitney U test was used to examine the difference in TVR between two groups, while Kruskal Wallis test was used to examine the difference in TVR between more than two groups

^b^A multivariate logistic regression analysis was performed to assess whether there were significant differences between age, KPS, and MGMTpm%. By using the median value of 1.20, TVR was categorized into binary dependent variable (1: better response with TVR ≤ 1.20; 0: poorer response with TVR > 1.20)

### Pre-treatment RTV and TVR

The median pre-treatment residual tumor volume (RTV) was 2511 mm^3^ (IQR 953–5927). When patients were categorized into two groups according to median pre-treatment RTV, no significant differences in MGMTpm% (p = 0.2693 by Mann–Whitney U test) and treatment response: CR/PR or SD/PD (p = 0.7597 by two-sided Fisher’s exact test), were observed between the two groups. In case of smaller pre-treatment RTV, 8 (72.7%) of 11 patients with MGMTpm% ≥ 10%, and 2 (18.2%) of 11 patients with MGMTpm% < 10% showed a tumor volume decrease of ≥ 50% (TVR ≤ 0.5). Similarly, in case of larger pre-treatment RTV, 5 (71.4%) of 7 patients with MGMTpm% ≥ 10%, and 2 (13.3%) of 15 patients with MGMTpm% < 10% showed a tumor volume decrease of ≥ 50% (TVR ≤ 0.5).

### MGMTpm% and TVR

**Fig. 1:**
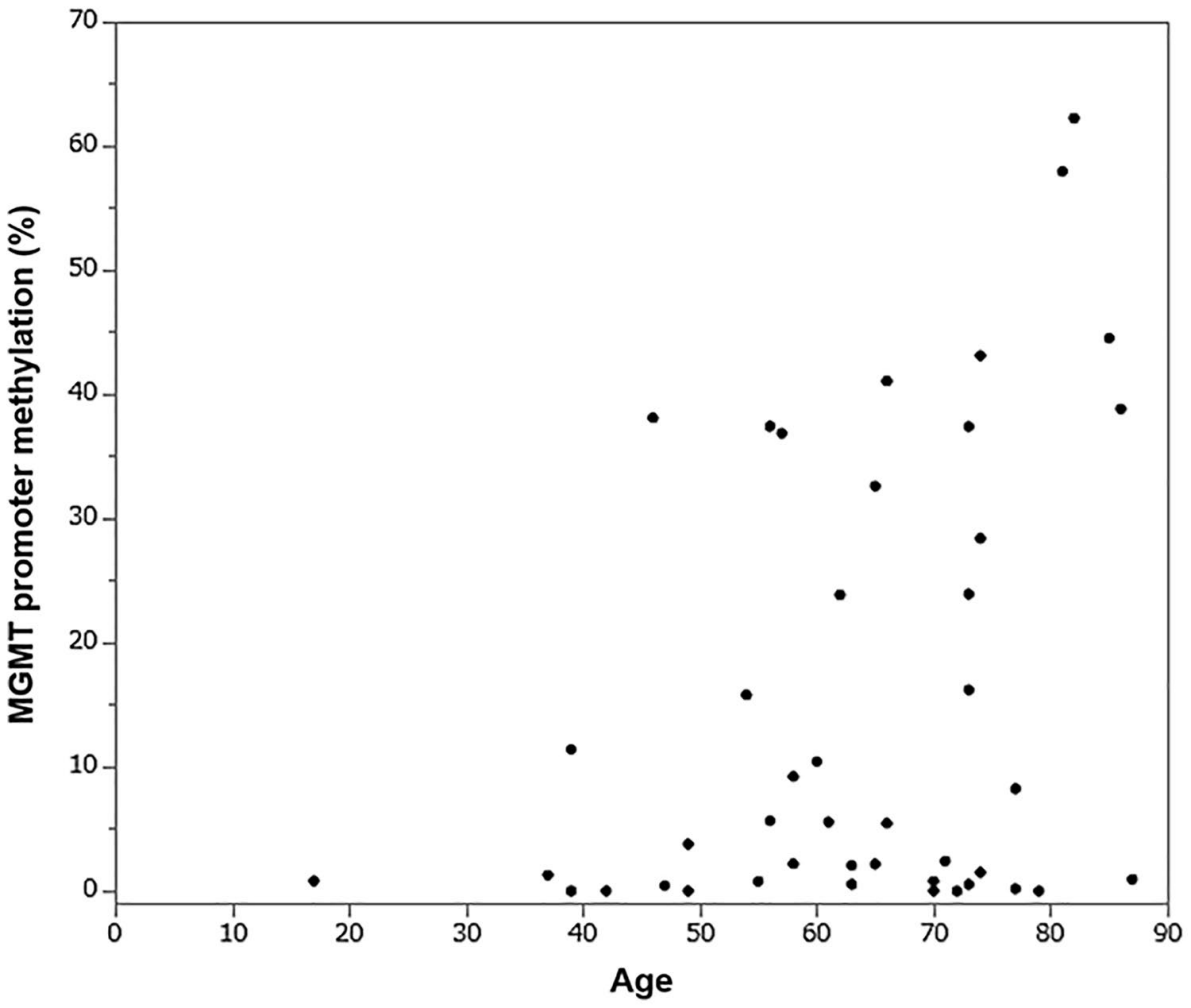
MGMT promoter methylation % of each patient is shown in relation to age

Figure 1 shows the distribution of MGMTpm% with age. Although there was no significant difference in MGMTpm% between patients aged ≥ 65 years and patients aged < 65 years (p = 0.2490 by Mann–Whitney U test), the scatter plot shows a trend that MGMTpm% increases with an increase in patient age (Fig. 1). Older patients had poorer KPS than younger patients (p = 0.0065 by two-sided Fisher’s exact test, data not shown).

**Fig. 2:**
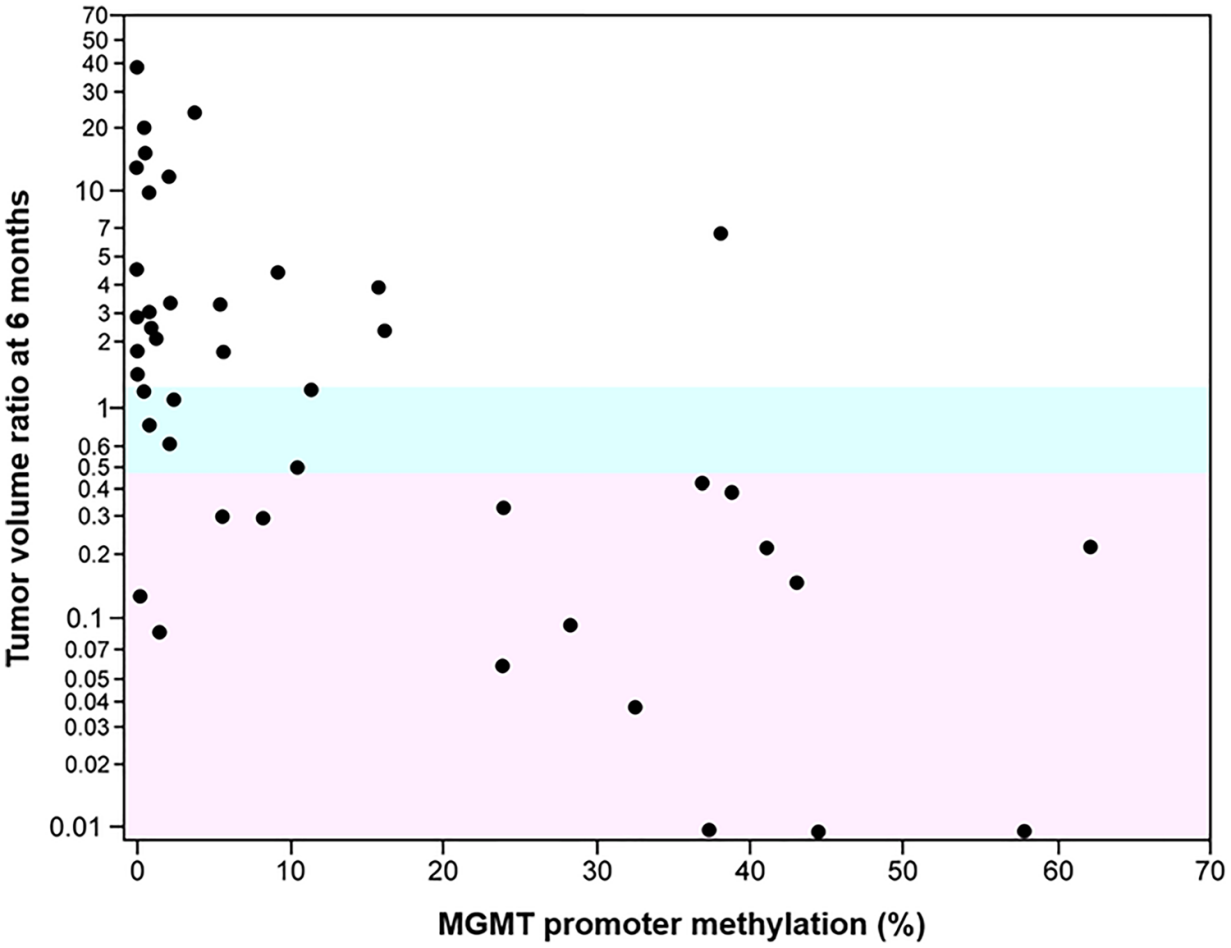
A scatter plot representation of tumor volume ratio (TVR) at 6 months after initial chemoradiotherapy administration in relation to MGMT promoter methylation %

Figure 2 shows a scatter plot representation of TVR at 6 months after the start of initial chemoradiotherapy in relation to MGMTpm%, which shows that TVR decreases with an increase in MGMTpm%. Thirteen (92.9%) of 14 patients with MGMTpm% ≥ 20% had a tumor volume decrease of ≥ 50%; however, 18 (69.2%) of 26 patients with MGMTpm% < 10% showed a tumor volume increase of ≥ 25%.

### MGMTpm% cutoff value

**Fig. 3:**
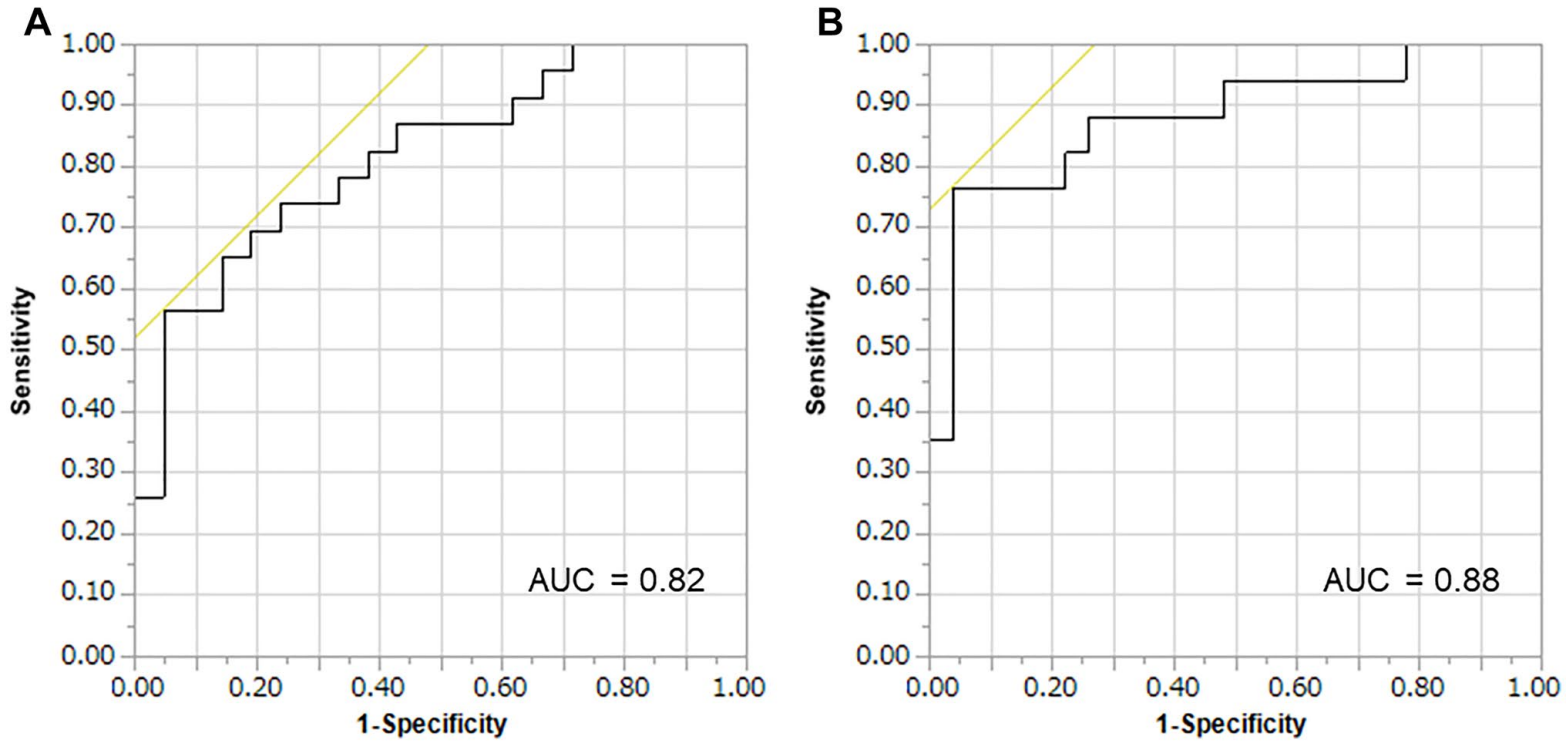
ROC curve for representative cutoff values of MGMT promoter methylation level. MGMT promoter methylation % were plotted with true positives on the vertical axis (sensitivity) and false positives (1-specificity) on the horizontal axis. **A** CR/PR/SD group versus PD group with TVR cutoff value of 1.25, and **B** CR/PR group versus SD/PD group with TVR cutoff value of 0.5

Given these findings, we analyzed the diagnostic value of MGMTpm% for TVR in response to temozolomide using ROC curve analysis**.** Figure 3 shows the ROC curve for the representative cutoff values of MGMTpm%. When MGMTpm% was 8.2% or more, there was a trend toward more cases that achieved SD or better (CR/PR/SD) at 6 months after initial chemoradiotherapy administration, with a diagnostic sensitivity of 70.0% and a specificity of 81.0% (Fig. 3A). When MGMTpm% was ≥ 23.9%, there was a trend toward more cases that achieved PR or better (CR/PR) at 6 months after initial chemoradiotherapy administration with a diagnostic sensitivity of 76.5% and a specificity of 96.3% (Fig. 3B). The population of MGMTpm% < 8.2%, 8.2% to < 23.9%, and ≥ 23.9% were 54.6%, 13.6%, and 31.8%, respectively (Table 1). Table 2A shows the relationship between MGMTpm% and volumetric response. Thirteen (92.9%) of 14 patients with MGMTpm% ≥ 23.9% showed CR/PR and 17 (70.8%) of 24 patients with MGMTpm% < 8.2% had PD.

**Table 2:** Relationship of MGMTpm% and response (A) or KPS (B), and TVR and change of KPS (C)

	MGMTpm%	Patients (%)	CR	PR	SD	PD
(A)	< 8.2%	24 (54.6%)	0 (0%)	3 (12.5%)	4 (16.7%)	17 (70.8%)
	8.2 to < 23.9%	6 (13.6%)	0 (0%)	1 (16.7%)	2 (33.3%)	3 (50.0%)
	≥ 23.9%	14 (31.8%)	4 (28.6%)	9 (64.3%)	0 (0%)	1 (7.1%)

	All	Patients (%)	Change of KPS			
			Improve	Stable	Deteriorate	
		44 (100%)	8 (18.2%)	15 (34.1%)	21 (47.7%)	
(B)	MGMTpm%					
	< 8.2%	24 (54.6%)	1 (4.2%)	8 (33.3%)	15 (62.5%)	
	8.2 to < 23.9%	6 (13.6%)	1 (16.7%)	2 (33.3%)	3 (50.0%)	
	≥ 23.9%	14 (31.8%)	6 (42.9%)	5 (35.7%)	3 (21.4%)	
(C)	TVR (response)					
	0 (CR)	4 (9.1%)	2 (50.0%)	1 (25.0%)	1 (25.0%)	
	< 50% (PR)	13 (29.6%)	5 (38.5%)	4 (30.8%)	4 (30.8%)	
	50 to < 125% (SD)	6 (13.6%)	0 (0%)	2 (33.3%)	4 (66.7%)	
	125% (PD)	21 (47.7%)	1 (4.8%)	8 (38.1%)	12 (57.1%)	

*MGMTpm%* MGMT promoter methylation %, *TVR* Tumor volume ratio, *KPS* Karnofsky performance status, *CR* complete response, *PR* partial response, *SD* stable disease, *PD* progressive disease

**Fig. 4:**
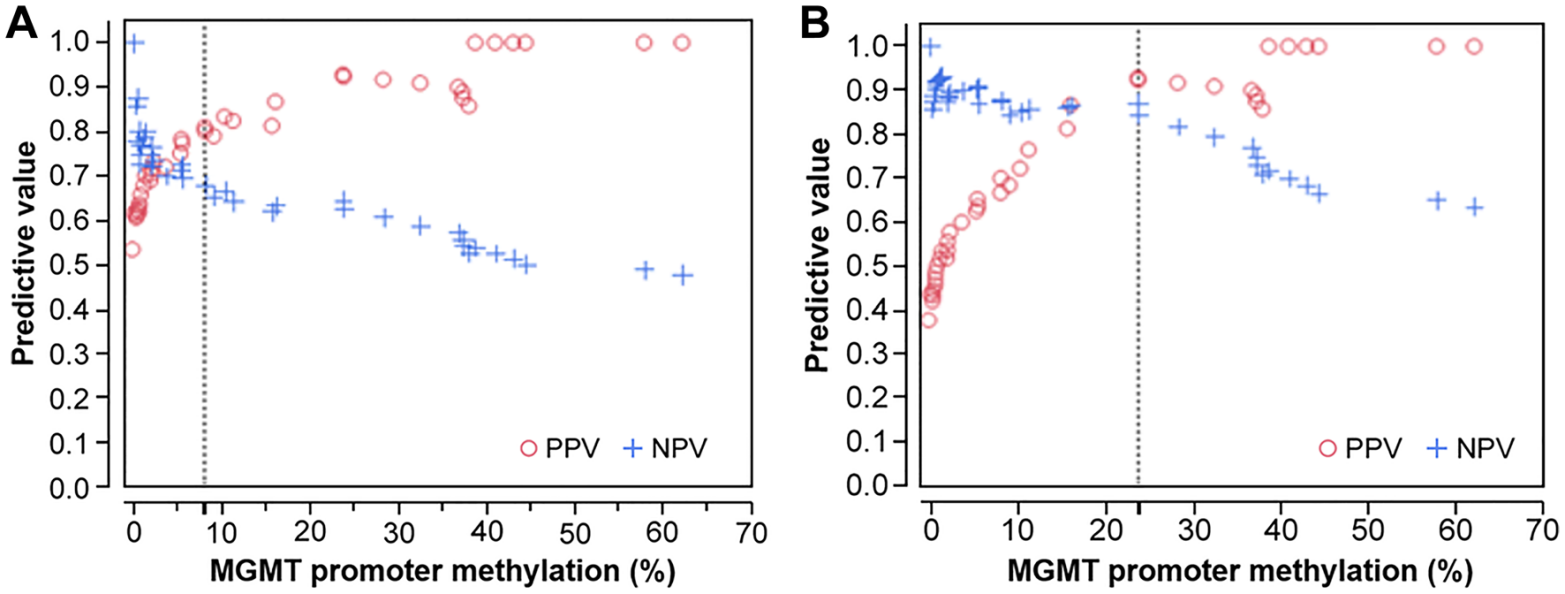
Positive predictive value (PPV) and negative predictive value (NPV) based on the ROC curve are shown. A: CR/PR/SD versus PD, and B: CR/PR versus SD/PD. Vertical axis shows predictive values and horizontal axis shows MGMT promoter methylation %

Figure 4 shows positive predictive value (PPV) and negative predictive value (NPV) based on ROC analysis as shown in Fig. 3. PPV indicates the probability that MGMT promoter high-methylated patients can achieve CR/PR/SD (Fig. 4A: a cutoff value of 8.2%) or CR/PR (Fig. 4B: a cutoff value of 23.9%) at 6 months after initial chemoradiotherapy administration. NPV indicates the probability that MGMT low-methylated patients fail to achieve CR/PR/SD (A: a cutoff value of 8.2%) or CR/PR (B: a cutoff value of 23.9%) at 6 months after initial chemoradiotherapy administration. By plotting PPV and NPV depending on MGMTpm%, we confirmed these cutoff values as reasonable (Fig. 4). Figure 4A showed approximately 80% of patients with MGMT promoter methylation ≥ 8.2% achieved CR/PR/SD and Fig. 4B showed more than 90% of patients with MGMT promoter methylation ≥ 23.9% achieved CR/PR.

### MGMTpm% and survival

**Fig. 5:**
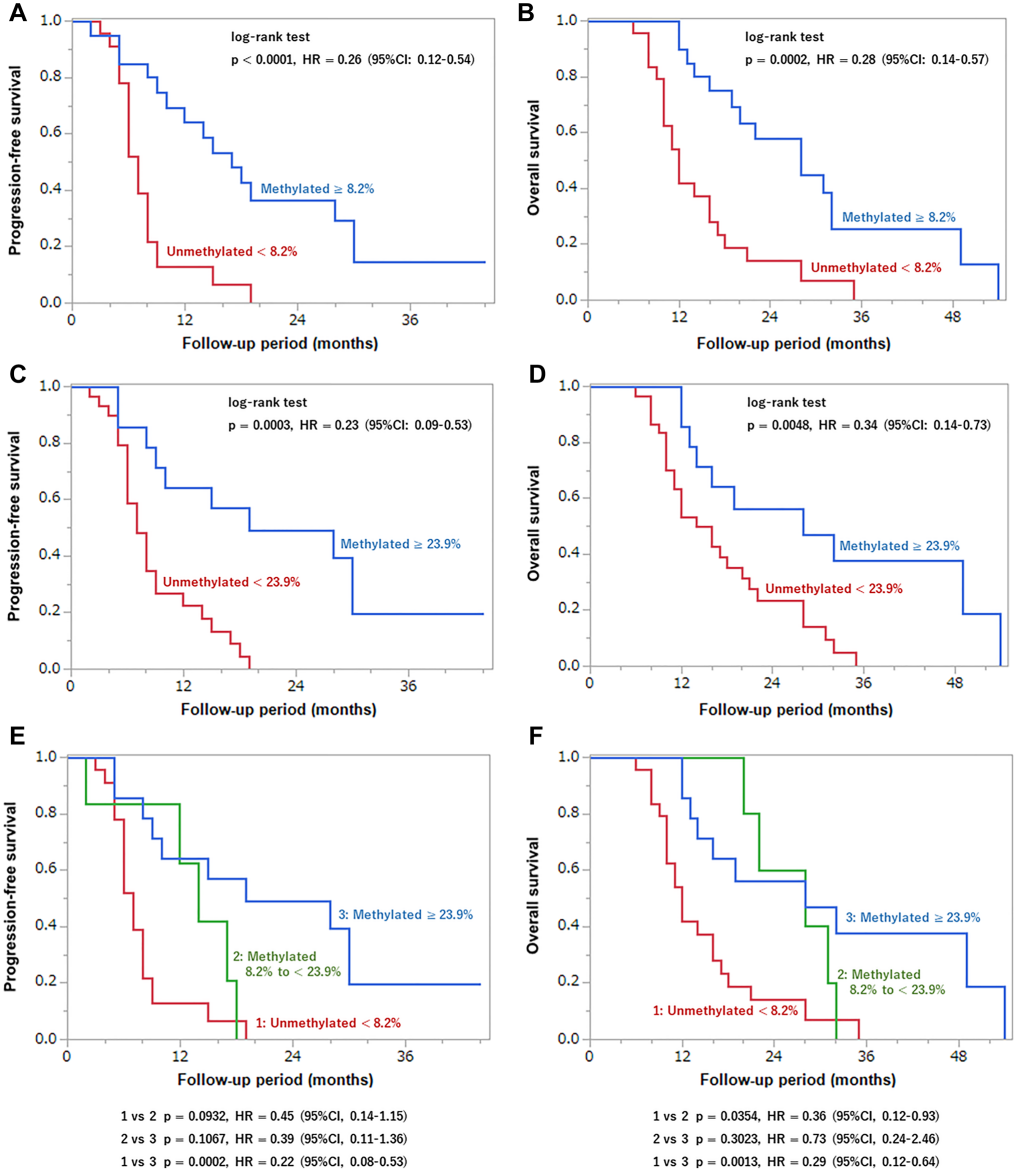
Kaplan–Meier curves for PFS and OS of GBM patients stratified by MGMT promoter methylation % cutoff value of 8.2% (**A** and **B**) and 23.9% (**C** and **D**). In **E** and **F**, the red, green, and blue lines indicate Group 1 (unmethylated < 8.2%), Group 2 (methylated 8.2% to < 23.9%), and Group 3 (methylated ≥ 23.9%), respectively. p values were calculated by log-rank test. *HR* hazard ratio, *CI* confidence interval

Figure 5 shows the OS and PFS with the cutoff values divided by 8.2% (Fig. 5A, B) or 23.9% (Fig. 5C, D) of MGMTpm% by the Kaplan–Meier method. The median PFS and OS in patients with MGMTpm% ≥ 8.2% were 16 and 24.5 months, respectively, and those of < 8.2% were 6.5 and 12 months, respectively. Patients with MGMTpm% ≥ 8.2% showed significantly longer PFS (HR 0.26, 95% CI 0.12–0.54, p < 0.0001) and OS (HR 0.28, 95% CI 0.14–0.57, p = 0.0002) than those with < 8.2%. Median PFS and OS in patients with ≥ 23.9% MGMTpm% were 18.5 and 23 months, respectively, and those with < 23.9% were 7 and 13.5 months, respectively. Patients with MGMTpm% ≥ 23.9% showed significantly longer PFS (HR 0.23, 95% CI 0.09–0.53, p = 0.0003) and OS (HR 0.34, 95% CI 0.14–0.73, p = 0.0048) than those with < 23.9%. Additionally, we stratified patients into three groups with MGMT cutoff values of 8.2% and 23.9%, namely (Group 1) < 8.2% of MGMTpm%, (Group 2) 8.2% to < 23.9%, and (Group 3) ≥ 23.9%, and showed the OS and PFS in each group. Group 2 showed a significantly longer OS (HR 0.36, 95% CI 0.12–0.93, p = 0.0354) than Group 1. Group 3 also showed significantly longer PFS (HR 0.22, 95% CI 0.08–0.53, p = 0.0002) and OS (HR 0.29, 95% CI 0.12–0.64, p = 0.0013) than Group 1. However, there was no significant difference in PFS (HR 0.39, 95% CI 0.11–1.36, p = 0.1067) and OS (HR 0.73, 95% CI 0.24–2.46, p = 0.3023) between Group 2 and Group 3.

### MGMTpm% and KPS

Table 2 shows the relationship between MGMTpm% (B) and tumor response (C) and the change in KPS. Six (42.9%) of 14 patients with MGMTpm% ≥ 23.9% improved KPS, but 15 (62.5%) of 24 patients with MGMTpm% < 8.2% had worse KPS. Seven (41.2%) of 17 patients with CR/PR improved KPS, but only one (3.7%) of 27 patients with SD/PD improved KPS. Furthermore, 5/17 (29.4%) patients with CR/PR worsened KPS and 16/27 (59.3%) patients with SD/PD deteriorated KPS. Thirteen (76.5%) of 17 patients with CR/PR and 18 (66.7%) of 27 patients with SD/PD maintained KPS ≥ 70, while 4/17 (23.5%) patients with CR/PR and 9/27 (33.3%) patients with SD/PD had a KPS < 70.

## Discussion

In this study, we analyzed the relationship between the volumetric tumor response and MGMTpm% by pyrosequencing method. Our data showed that 92.9% of patients with MGMTpm% ≥ 23.9% decreased tumor volume by more than 50% (CR/PR) at 6 months after the start of chemoradiotherapy, and 42.9% of those patients with high MGMTpm% improved KPS, leading to a good prognosis. However, 70.8% of patients with MGMTpm% < 8.2% showed PD, and 62.5% of those patients with low-MGMTpm% had worse KPS. These predictions are important in explaining the prognosis to the patient. We also analyzed the relationship between pre-treatment RTV, MGMTpm%, and TVR. No significant relationship was found between pre-treatment RTV and TVR; higher MGMTpm% could lead to greater tumor volume decrease, even if the pre-treatment RTV was small or large.

Although the usefulness of MGMT promoter methylation analysis in glioblastoma patients for predicting the prognosis and response to chemoradiotherapy with temozolomide has been widely reported, there is still no consensus regarding how to measure MGMTpm% and how to define MGMTpm% cutoff values. Since glioblastoma patients without MGMT promoter methylation are resistant to temozolomide and have a worse prognosis, the treatment strategy depends on MGMT promoter methylation status. Wick et al. reported that event-free survival was longer in patients with MGMT promoter methylation who received temozolomide alone than in those who underwent radiotherapy alone in older patients [13]. A phase 3 CheckMate-498 study comparing radiotherapy with temozolomide and radiotherapy with nivolumab did not meet the primary endpoint of overall survival in patients with newly diagnosed MGMT-unmethylated glioblastoma patients [14]. Additionally, temozolomide was reported to prolong PFS and OS even in patients who were diagnosed with MGMT promoter unmethylation status [2]. It is possible that the patients who were diagnosed with absence of MGMT promoter methylation and were treated with radiotherapy with nivolumab did not benefit from temozolomide treatment. Therefore, the diagnosis of MGMT promoter methylation status is important for selecting patients, especially in clinical trials.

Currently, representative methods to analyze MGMT promoter methylation status include methylation-specific PCR (MSP) and pyrosequencing methods [15]. Since MSP [16] can only assess the methylation status of two regions (CpG76–80 and CpG84–87), the method might be disadvantageous in that only qualitative evaluation of methylation status in relatively limited regions is possible. Lattanzio et al. reported that the reliability of the MSP method was inferior to that of the pyrosequencing method when the methylation status was heterogeneous in each CpG region within individuals [17]. Therefore, the pyrosequencing method might have relatively better specificity and sensitivity than the MSP method, and quantitative evaluation of methylation status in each CpG region is possible even in cases with methylation heterogeneity [15, 18–24].

There have been many reports that used pyrosequencing and analyzed the MGMT promoter methylation status cutoff value for predicting the prognosis of glioblastoma patients. However, since the MGMT promoter methylation status cutoff values may differ depending on the median OS values of the study samples used as criteria for stratification, the cutoff values used for prediction vary from 2.68 [20] to 35% [25]. Dunn et al. determined binary values of 29% as significant MGMTpm% cutoff values averaged in 12 CpG regions [25]. Brigliadori et al. classified MGMT promoter methylation in 10 CpG regions with binary cutoff values of 9% and 30%; only patients whose MGMTpm%s were > 30% had the predictive role of MGMT promoter methylation [18]. The Cochrane report analyzed many studies and concluded that their meta-analysis did not provide strong evidence about the best CpG sites or threshold, but that a cutoff threshold of 9% for CpG sites 74–78 performed better than higher thresholds of 28% or 29% [15].

Given the considerations mentioned above, we aimed to define the optimal MGMT promoter methylation cutoff value via statistical analysis focused on the TVR and determined the predictive indicators in a real-world clinical context. The survival-based analysis could be affected by treatments other than temozolomide which were administered after tumor recurrence, while the TVR-based analysis is more useful for directly predicting the temozolomide efficacy. With regard to treatment efficacy at 6 months after initial chemoradiotherapy, patients whose MGMTpm% were (1) 8.2% or more and (2) 23.9% or more had a propensity to achieve (1) SD or better (CR/PR/SD) and (2) PR or better (CR/PR), respectively. Thus, the binary values of 8.2% and 23.9% seemed to be useful as cutoff values. In this study, the OS was significantly prolonged in patients whose MGMTpm%s was 8.2% to < 23.9%, and patients whose MGMTpm%s was > 23.9% obtained significantly prolonged PFS and OS with the lowest p values. Subdividing MGMT promoter methylation groups with binary cutoff values may be useful for accurately predicting treatment efficacy and prognosis. Our study results can be more practical because the cutoff values were determined by the TVR rather than by the survival, which may vary depending on the population or sample, as previously reported [15, 17–21, 25].

The first step in the treatment of glioblastoma patients is maximal safe resection without neurological deterioration. However, more than half of the patients cannot undergo gross total resection, leading to poor prognosis. Our report showed that a patient with high MGMT promoter methylation status has the possibility of tumor shrinkage due to temozolomide and improvement of KPS. Intraoperative diagnosis of MGMT promoter methylation [26] or preoperative assessment by deep machine learning [27] provide useful information in surgical procedures.

This study was limited by its retrospective design and relatively small population size, and further validation studies are required to validate these results. The intratumoral variety of MGMTpm% might have also influenced these results. Therefore, future large-scale studies are warranted.

In conclusion, the evaluation of MGMTpm% by pyrosequencing is important in predicting volumetric change, change in KPS and prognosis of glioblastoma patients with residual tumors.

## Data Availability

The data in this study are available from the corresponding author on reasonable request.
